# Genistein Induces Adipogenic and Autophagic Effects in Rainbow Trout (*Oncorhynchus mykiss*) Adipose Tissue: In Vitro and In Vivo Models

**DOI:** 10.3390/ijms21165884

**Published:** 2020-08-16

**Authors:** Sara Balbuena-Pecino, Esmail Lutfi, Natàlia Riera-Heredia, Esther Gasch-Navalón, Emilio J. Vélez, Joaquim Gutiérrez, Encarnación Capilla, Isabel Navarro

**Affiliations:** Departament de Biologia Cellular, Fisiologia i Immunologia, Facultat de Biologia, Universitat de Barcelona, 08028 Barcelona, Spain; sara.balbuena@ub.edu (S.B.-P.); esmailroyo@gmail.com (E.L.); natalia.riera@gmail.com (N.R.-H.); esthergasch19@gmail.com (E.G.-N.); evelezve@gmail.com (E.J.V.); jgutierrez@ub.edu (J.G.); ecapilla@ub.edu (E.C.)

**Keywords:** genistein, 17β-estradiol, phytoestrogens, lipid metabolism, autophagy, apoptosis

## Abstract

Soybeans are one of the most used alternative dietary ingredients in aquafeeds. However, they contain phytoestrogens like genistein (GE), which can have an impact on fish metabolism and health. This study aimed to investigate the in vitro and in vivo effects of GE on lipid metabolism, apoptosis, and autophagy in rainbow trout (*Oncorhynchus mykiss*). Primary cultured preadipocytes were incubated with GE at different concentrations, 10 or 100 μM, and 1 μM 17β-estradiol (E2). Furthermore, juveniles received an intraperitoneal injection of GE at 5 or 50 µg/g body weight, or E2 at 5 µg/g. In vitro, GE 100 μM increased lipid accumulation and reduced cell viability, apparently involving an autophagic process, indicated by the higher LC3-II protein levels, and higher *lc3b* and *cathepsin d* transcript levels achieved after GE 10 μM. In vivo, GE 50 µg/g upregulated the gene expression of fatty acid synthase (*fas*) and glyceraldehyde-3-phosphate dehydrogenase in adipose tissue, suggesting enhanced lipogenesis, whereas it increased hormone-sensitive lipase in liver, indicating a lipolytic response. Besides, autophagy-related genes increased in the tissues analyzed mainly after GE 50 µg/g treatment. Overall, these findings suggest that an elevated GE administration could lead to impaired adipocyte viability and lipid metabolism dysregulation in rainbow trout.

## 1. Introduction

The rapid growth of aquaculture in the last years and the subsequent increase in aquafeeds production have resulted in high demand for fishmeal and fish oil (FO). Thus, reducing their use and developing new fish diets with alternative nutrient sources are nowadays recognized as a priority for the future growth and sustainability of the industry [1]. In this regard, although vegetable oils are relatively poor sources of n-3 fatty acids in comparison to FO [2], they have been incorporated as alternative ingredients in fish diets with successful results [3,4,5,6]. Among them, soybean oil has been one of the most used as a non-fish source of n-6 and n-3 polyunsaturated fatty acids [2,7,8]. 

Soy products are used in human nutrition due to their beneficial health effects. They lower the risk factors for cardiovascular diseases by decreasing triglycerides (TAG), total cholesterol, and low-density lipoprotein blood levels [9]. Moreover, it has been postulated that some soy compounds might have antioxidant [10,11] and anti-cancer properties [12,13]. Although these studies provided key information about their valuable application in human healthcare, soy-foods have become controversial in recent years. Of any plant, soy contains the highest concentration of isoflavones, mainly genistein (GE) and daidzein, a class of phytoestrogens. Phytoestrogens are biologically active molecules that are structurally similar to 17β-estradiol (E2). This resemblance enables them to bind estrogen receptors and mimic E2 effects. However, both estrogenic and anti-estrogenic effects have been reported in mammals depending on the model and parameter evaluated. For example, considering the effects on adipose tissue, GE inhibited leptin secretion independently of estrogen receptor activation in murine adipocytes, while the inhibition of adipogenesis in human adipose tissue-derived mesenchymal stem cells by GE was estrogen receptor-dependent (reviewed in [14,15]).

These isoflavones may have positive but also negative effects in health, through impacting lipid metabolism, which is often accompanied by alterations in the hormonal status of the organism [16]. In vitro mammalian studies have shown that GE impairs adipocyte differentiation in the 3T3-L1 cell line, leading to apoptosis of mature adipocytes [17], and reduces lipid accumulation in a dose-dependent manner in primary human preadipocytes, showing the cells exposed to high doses of GE a fibroblast-like morphology instead of the characteristic round shape of mature adipocytes [18]. Regarding in vivo assays, GE is generally considered hypolipidemic [19], but differences have been shown depending on its content in the diet. A reduction in adipose tissue mass has also been reported in several in vivo mice models with GE stimulating lipolysis and adipocytes apoptosis at the same time (reviewed in [14]), while in human visceral preadipocytes, GE has been demonstrated to promote adipogenesis [20]. In addition, high levels of dietary GE included in mice diets decreased hepatic fat accumulation by increasing fatty acid oxidation, and even a low dose of GE increased mitochondrial enzyme activity in mice with fatty liver and obesity induced by high-fat diets, although specific effects on adipose tissue were not mentioned in that study [21]. Moreover, it has been recently shown that GE can also influence autophagy, the cellular degradation process by which cytoplasmic components are degraded in the lysosomes [22]. The induction of autophagy as a result of GE exposure has been documented in human breast MCF-7 cells [18] and several ovarian cancer cell lines [19]. 

The specific effect of GE and its mechanism of action on metabolically active tissues in fish like liver or white muscle have been studied; nevertheless, the adipose tissue still remains largely unexplored. In this sense, in vivo administration and in vitro studies have shown that GE affects muscle protein turnover by increasing the rates of protein degradation and proteolysis-related genes expression in rainbow trout (*Oncorhynchus mykiss*), via estrogen receptor-dependent and independent mechanisms [23]. The ability of GE to produce an estrogenic effect has also been reported in Atlantic salmon (*Salmo salar*) hepatocyte cells, through the increase in *vitellogenin* (*vtg*) mRNA levels [24]. Besides, GE downregulated growth hormone/insulin-like growth factor axis-related genes and promoted changes in several other genes’ expression, inducing a dysregulation of lipid metabolism in the liver [25]. In sea bass (*Dicentrarchus labrax*) scales exposed to GE, increased enzymatic activities (alkaline phosphatase and tartrate-resistant acid phosphatase), indicative of a higher mineral turnover, were reported, as well as a similar response of GE and E2 in scale and liver gene expression [26]. Moreover, in the early life stages of Senegalese sole (*Solea senegalensis*), GE exposure led to normal larval development and growth, but evidence of induced apoptosis was not observed [27]. Apart from these research works, and as far as we know, the effects of GE in fish adipose tissue have not been elucidated yet.

In this framework, the main aim of the present study was to investigate in rainbow trout the effects of two doses of GE on lipid metabolism, apoptosis, and autophagy using in vitro and in vivo approaches. We hypothesized, based on mammalian literature, that GE could potentially act as an anti-adipogenic compound in rainbow trout when administered at an adequate level.

## 2. Results

### 2.1. In Vitro Evaluation of GE Effects on Primary Cultured Adipocytes

#### 2.1.1. Adipocyte Cells Viability, Nuclear Morphology, and LC3-II Protein Expression 

**Figure 1:**
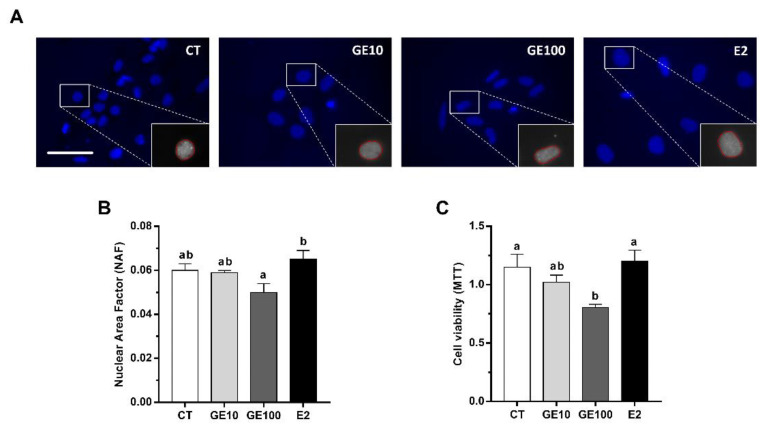
In vitro effects on pre-confluent rainbow trout preadipocytes incubated at day 5 of culture for 24 h with GE at different concentrations (10 and 100 μM), E2 (1 μM), or vehicle (0.1% DMSO) as CT. (**A**) Representative immunofluorescence images stained with Hoechst for nuclei and (**B**) quantification of the nuclear area factor (NAF). (**C**) Quantification of cell viability using a methylthiazolyldiphenyl-tetrazolium bromide (MTT) assay. Scale bar 100 μm. Data are shown as mean + SEM (*n* = 4–6). Significant differences among treatments were determined by one-way ANOVA and are indicated by different letters (*p* < 0.05). When two groups share at least one letter, they are not statistically different. CT: Control; GE: Genistein; E2: 17β-estradiol.

**Figure 2:**
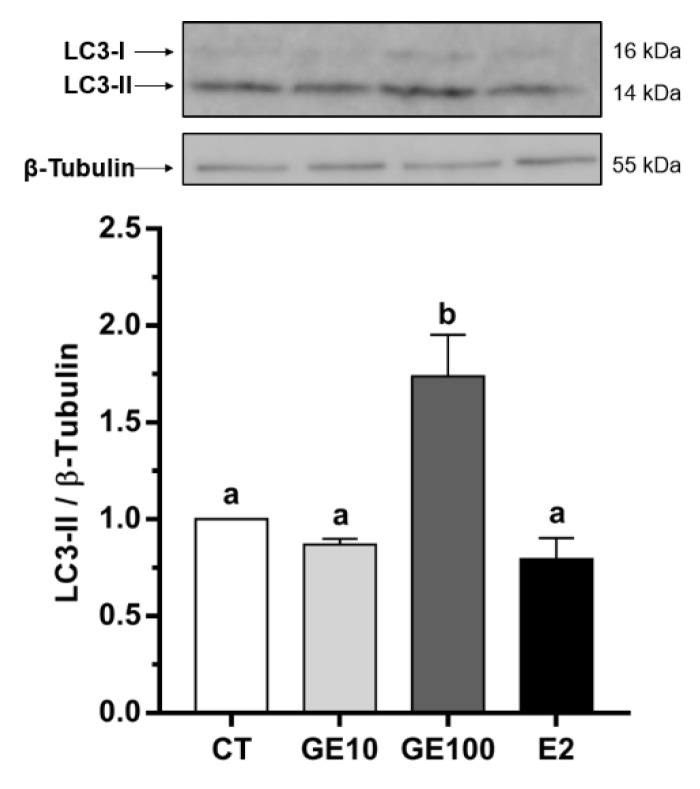
In vitro effects on pre-confluent rainbow trout preadipocytes incubated at day 5 of culture for 24 h with GE at different concentrations (10 and 100 μM), E2 (1 μM), or vehicle (0.1% DMSO) as CT. Representative Western blots and quantification of LC3-II protein levels normalized to β-tubulin. Data are shown as mean + SEM (*n* = 4–6). Significant differences among treatments were determined by one-way ANOVA and are indicated by different letters (*p* < 0.05). CT: Control; GE: Genistein; E2: 17β-estradiol.

Changes in nuclear morphology, as an early sign of apoptosis [28], in response to the different treatments were observed. Generally, the nuclei from the cells under the different treatments were rounded, while, in the case of GE 100 μM-treated cells, some of them were less circular or even showed an elongated shape (Figure 1A). Indeed, in the quantitative analysis, the GE 100 μM treatment was found to affect nuclear morphology, showing a reduction in nuclear area factor (NAF). However, differences were only significant with respect to E2-exposed adipocytes, which, in turn, showed slightly but not significantly higher NAF values than control (CT) cells (Figure 1B). In fact, GE 10 μM and E2 did not induce significant changes in cell viability compared to CT cells; however, GE at 100 μM significantly reduced cell viability values compared to the CT and E2 groups (Figure 1C). Concerning the autophagy marker, microtubule-associated protein-1 light chain 3b (LC3b), significantly higher protein expression levels of the lipidated form, LC3-II, were found in adipocytes after GE 100 μM treatment compared to all the other conditions (Figure 2). 

#### 2.1.2. Adipocyte Gene Expression Related to Apoptosis and Autophagy 

**Figure 3:**
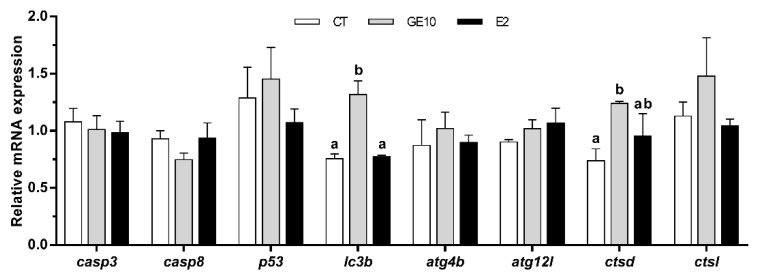
In vitro effects on confluent rainbow trout preadipocytes incubated at day 7 of culture for 72 h with GE (10 μM), E2 (1 μM), or vehicle (0.1% DMSO) as CT over the expression of apoptosis- and autophagy-related genes. Relative mRNA expression normalized to *ef1α* and *β-actin* of *casp3*, *casp8*, *p53*, *lc3b*, *atg4b*, *atg12l*, *ctsd*, and *ctsl*. Data are shown as mean + SEM (*n* = 6). Significant differences among treatments for each gene were determined by one-way ANOVA and are indicated by different letters (*p* < 0.05). When two groups share at least one letter, they are not statistically different. CT: Control; GE: Genistein; E2: 17β-estradiol.

To further characterize the in vitro effects of GE over the possible activation of apoptosis and autophagy processes, we analyzed the gene expression of several markers from these two pathways after a 72 h treatment. Nevertheless, only the results from GE 10 μM-treated cells are shown as the dose of GE 100 μM caused a significant cell death to the cells and, as a result, we could not extract enough RNA from these samples to perform the analysis. The quantitative expression of genes implicated in apoptosis analyzed in primary cultured adipocytes did not reveal any differences among groups. Concerning the autophagy markers, GE 10 μM significantly enhanced the mRNA levels of *lc3b* and cathepsin d (*ctsd*) compared to CT cells, and in the case of the former, also with respect to E2-treated cells. In addition, cathepsin l (*ctsl*) presented a tendency to be higher after GE exposure, although this was not significant. Differences were not observed for autophagy-related 4b cysteine peptidase (*atg4b*) and autophagy-related gene 12-like (*atg12l*) (Figure 3).

#### 2.1.3. Adipocyte Differentiation, Lipid Accumulation, and Released Metabolites

**Figure 4:**
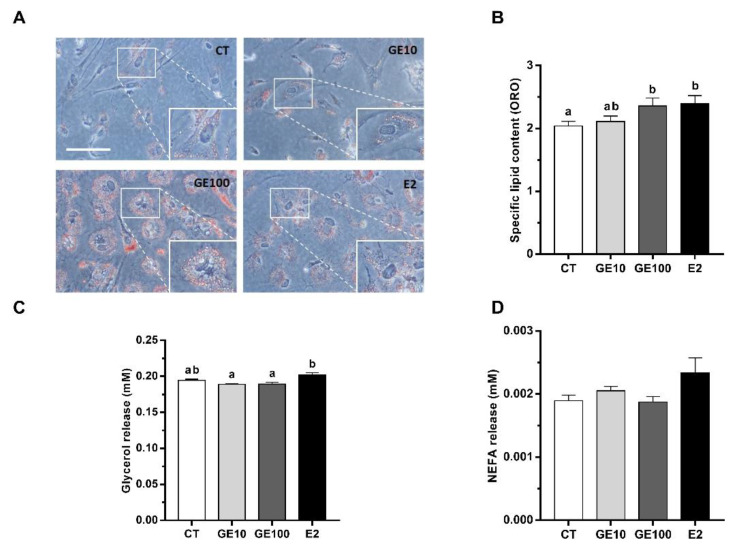
In vitro effects on confluent rainbow trout preadipocytes incubated at day 7 of culture for 72 h with GE at different concentrations (10 and 100 μM), E2 (1 μM), or vehicle (0.1% DMSO) as CT. (**A**) Representative phase-contrast images of cells after staining with ORO and (**B**) quantification of lipid content. (**C**) Glycerol and (**D**) NEFA culture media levels. Scale bar 100 μm. Data are shown as mean + SEM (*n* = 4–6). Significant differences among treatments were determined by one-way ANOVA and are indicated by different letters (*p* < 0.05). When two groups share at least one letter, they are not statistically different. CT: Control; GE: Genistein; E2: 17β-estradiol; ORO: Oil red O; NEFA: Non-esterified fatty acids.

The images taken after Oil Red O (ORO) staining of cultured adipocytes showed a rounder shape and higher formation of intracellular lipid droplets in cells exposed to GE 100 μM and E2 than in CT and GE 10 μM groups (Figure 4A), which are both characteristics of the mature adipocyte phenotype. Quantification of specific lipid content confirmed image observation, as the exposure of rainbow trout adipocytes to GE 100 μM and E2 significantly increased the lipid content in the cells in comparison to the CT condition (Figure 4B). Moreover, the glycerol levels in the culture media were significantly higher in E2-treated cells compared to the cells incubated with the two doses of GE, but not to the CT group (Figure 4C). By contrast, released non-esterified fatty acids (NEFA) levels did not reveal differences among groups (Figure 4D).

### 2.2. In Vivo Evaluation of GE Effects after an Intraperitoneal Injection

#### 2.2.1. Plasma Metabolites and Hepatic Gene Expression of a Biomarker of Estrogen’s Exposure

Plasma levels of metabolites in fish administered with GE and E2 are shown in Table 1. TAG plasma levels in GE 50 μg/g-injected fish were significantly lower compared to the other three groups. In addition, the administration of E2 caused a significant increase in NEFA plasma concentration. Glycerol and glucose levels remained unaltered upon treatments.

Besides, gene expression of *vtg*, an identified biomarker of estrogen’s exposure, was evaluated in the liver and found significantly upregulated in the GE 50 μg/g and E2-injected fish compared to the other two groups (Table 2).

#### 2.2.2. Gene Expression Related to Lipid Metabolism, Apoptosis, and Autophagy in Adipose Tissue

**Figure 5:**
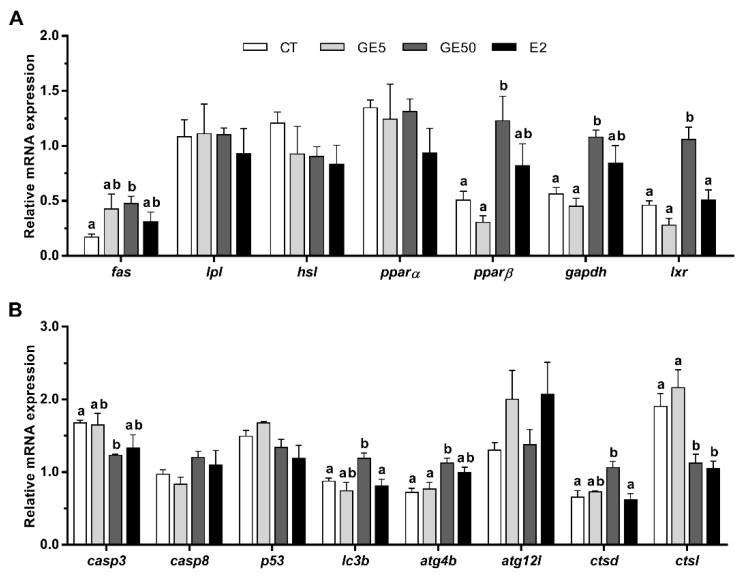
In vivo effects on rainbow trout intraperitoneally injected with vehicle (DMSO diluted 1:3 in sesame oil) as CT, GE 5 μg/g body weight, GE 50 μg/g, or E2 5 μg/g over the expression of lipid metabolism, and apoptosis- and autophagy-related genes in adipose tissue. Relative mRNA expression normalized to *ef1α* and *β-actin* of (**A**) *fas*, *lpl*, *hsl*, *pparα*, *pparβ*, *gapdh*, and *lxr*, and (**B**) *casp3*, *casp8*, *p53*, *lc3b*, *atg4b*, *atg12l*, *ctsd*, and *ctsl*. Data are shown as mean + SEM (*n* = 6–9). Significant differences among treatments for each gene were determined by one-way ANOVA and are indicated by different letters (*p* < 0.05). When two groups share at least one letter, they are not statistically different. CT: Control; GE: Genistein; E2: 17β-estradiol.

The de novo fatty acid synthesis of enzyme fatty acid synthase (*fas*) was significantly upregulated in rainbow trout with the high dose of GE compared to the CT group. Similarly, fish administered with GE at 50 μg/g significantly increased the gene expression of peroxisome proliferator-activated receptor β (*pparβ*), glyceraldehyde-3-phosphate dehydrogenase (*gapdh*), and liver x receptor (*lxr*) with respect to the CT animals and those treated with the low dose of GE, and in the case of *lxr*, also in comparison to the E2-injected fish. Contrarily, mRNA levels of the lipoprotein lipase (*lpl*), the hormone-sensitive lipase (*hsl*)*,* and the transcription factor *pparα* did not show differences under the experimental conditions tested (Figure 5A). 

On the other hand, following GE 50 μg/g administration, caspase 3 (*casp3*) mRNA levels significantly decreased compared to the CT group, while expression of *lc3b* and *ctsd* increased in comparison to CT and E2, and *atg4b* compared to CT and GE 5 μg/g. The mRNA levels of *ctsl* were significantly downregulated in the GE 50 μg/g and E2-injected fish compared to the other two groups. Aside from that, changes were not found in the expression of the other apoptosis-related genes, *casp8* and tumor protein p53 (*p53*), nor in *atg12l* upon treatments (Figure 5B). 

#### 2.2.3. Gene Expression Related to Lipid Metabolism, Apoptosis, and Autophagy in Liver and White Muscle

**Figure 6:**
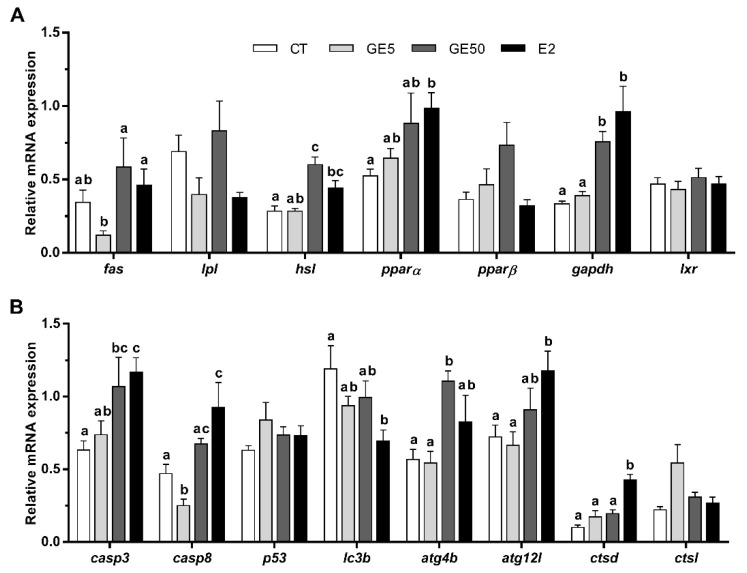
In vivo effects on rainbow trout intraperitoneally injected with vehicle (DMSO diluted 1:3 in sesame oil) as CT, GE 5 μg/g, GE 50 μg/g, or E2 5 μg/g over the expression of lipid metabolism, and apoptosis- and autophagy-related genes in liver. Relative mRNA expression normalized to *ubq* and *β-actin* of (**A**) *fas*, *lpl*, *hsl*, *pparα*, *pparβ*, *gapdh*, and *lxr*, and (**B**) *casp3*, *casp8*, *p53*, *lc3b*, *atg4b*, *atg12l*, *ctsd*, and *ctsl*. Data are shown as mean + SEM (*n* = 6–9). Significant differences among treatments for each gene were determined by one-way ANOVA and are indicated by different letters (*p* < 0.05). When two groups share at least one letter, they are not statistically different. CT: Control; GE: Genistein; E2: 17β-estradiol.

In the liver, the transcript levels of *fas* were significantly downregulated but only in GE 5 μg/g-injected juveniles compared to those fish treated with the high dose of GE or E2. Concerning lipases, *lpl* remained unaltered, but the lipolysis-associated gene *hsl* showed the highest levels of expression in rainbow trout administered with the GE 50 μg/g dose compared to the CT group. The mRNA levels of *ppar*α and *pparβ* showed a tendency to gradually increase in a dose-dependent manner in the animals treated with GE, although not significantly, while E2 injection significantly upregulated *ppar*α levels in comparison to the CT animals. Gene expression of *gapdh* was significantly upregulated in the GE 50 μg/g and E2-injected fish compared to the other two groups (Figure 6A).

Transcriptional levels of *casp3* in the liver were significantly higher after GE 50 μg/g and E2 injection. In addition, the gene expression of *casp8* was significantly downregulated by the low dose of GE with respect to the other three conditions, although increased in E2-injected fish in comparison to the CT. The expression of *p53* remained unaltered. Concerning the autophagic genes analyzed, a significant decrease in *lc3b* was observed with E2. By contrast, the high dose of GE caused an upregulation in the gene expression of *atg4b* versus the CT and the low-dose GE groups. Moreover, *atg12l* and *ctsd* mRNA levels after E2 exposure were the highest. Gene expression of *ctsl* showed a tendency to increase with GE 5 μg/g, although not significantly (Figure 6B). 

**Figure 7:**
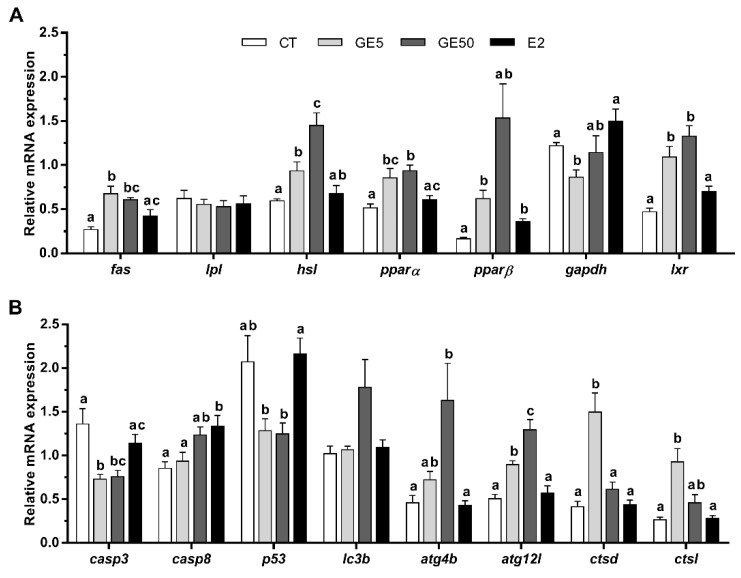
In vivo effects on rainbow trout intraperitoneally injected with vehicle (DMSO diluted 1:3 in sesame oil) as CT, GE 5 μg/g, GE 50 μg/g, or E2 5 μg/g over the expression of lipid metabolism, and apoptosis- and autophagy-related genes in white muscle. Relative mRNA expression normalized to *ubq* and *ef1α* of (**A**) *fas*, *lpl*, *hsl*, *pparα*, *pparβ*, *gapdh*, and *lxr*, and (**B**) *casp3*, *casp8*, *p53*, *lc3b*, *atg4b*, *atg12l*, *ctsd,* and *ctsl*. Data are shown as mean + SEM (*n* = 6–9). Significant differences among treatments for each gene were determined by one-way ANOVA and are indicated by different letters (*p* < 0.05). When two groups share at least one letter, they are not statistically different. CT: Control; GE: Genistein; E2: 17β-estradiol.

In white muscle, both doses of GE upregulated the mRNA levels of *fas*, compared to CT fish, although only the low dose caused a significant difference with the E2 group. As observed in both adipose tissue and liver, the gene expression levels of *lpl* were not affected upon treatments, while the mRNA levels of *hsl* gradually increased along with the GE dose. Regarding the transcription factors, *pparα* and *pparβ* showed significantly higher expression levels after the GE treatments compared to the CT, except for *pparβ* in the GE 50 μg/g-injected fish that was not significant. *pparβ* mRNA levels in E2-treated animals were also higher than those in the CT group. Moreover, the administration of GE 5 μg/g significantly decreased *gapdh* levels in comparison to CT and E2 groups. In addition, both doses of GE upregulated the transcript levels of *lxr* compared to CT and E2-treated fish (Figure 7A). 

Regarding apoptosis and autophagy, a downregulation of *casp3* with respect to CT fish was induced, in this case, after both doses of GE. On the other hand, fish exposed to E2 significantly increased the gene expression of *casp8* compared to CT and GE 5 μg/g groups, and increased p53 expression in comparison to both groups of fish treated with GE. Gene expression of *atg4b* and *atg12l* was significantly increased in rainbow trout after GE 50 μg/g exposure and in both GE groups, respectively, in comparison to CT and E2 groups. Finally, the administration of GE 5 μg/g significantly upregulated *ctsd* with respect to the other three groups and *ctsl* compared to CT and E2 groups (Figure 7B).

#### 2.2.4. Comparative and Qualitative Gene Expression Analysis among Tissues

**Figure 8:**
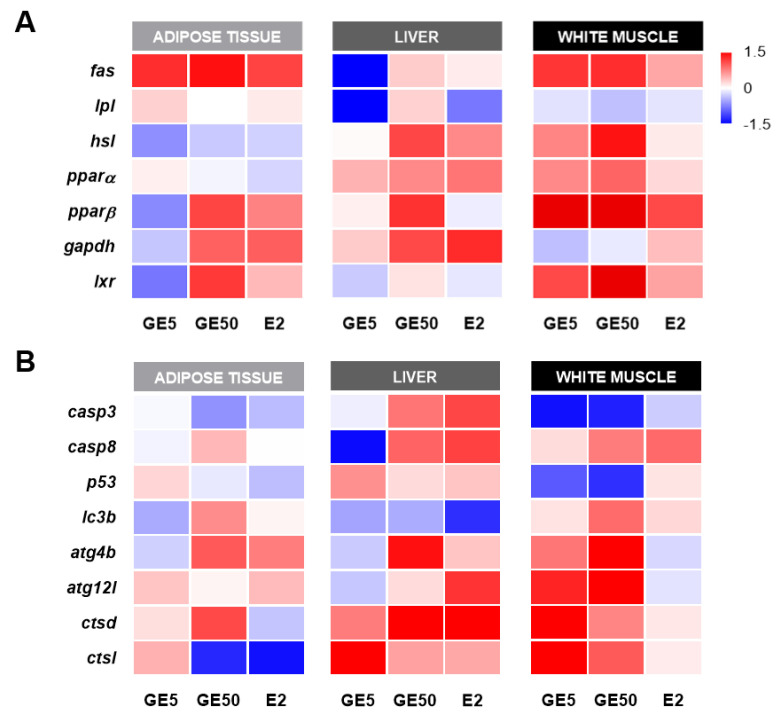
Comparative heat maps showing the changes in (**A**) lipid metabolism and (**B**) apoptosis- and autophagy-related genes expression among adipose tissue, liver, and white muscle in rainbow trout intraperitoneally injected with GE 5 μg/g, GE 50 μg/g, or E2 5 μg/g. Gene expression was first calculated relative to the geometric mean of the corresponding reference genes for each tissue, and then it was standardized following a standard score normalization (log2) against the corresponding control (CT) samples. Shades of red and blue, respectively, indicate the highest and lowest expression levels, as specified in the scale bar of the figure. GE: Genistein; E2: 17β-estradiol.

Finally, to do a comparative study among tissues of the effects on gene expression, all treatments were analyzed relative to their corresponding CT samples (Figure 8). Different expression patterns were seen depending on the tissue and the group of genes studied. Visual interpretation of the heatmap figures emphasizes a differential response upon GE doses in adipose tissue and liver, whereas, in white muscle, both treatments regulated most of the genes studied in a very similar manner. Particularly, with regard to lipid metabolism, both doses of GE upregulated (red) most of the genes analyzed in white muscle. By contrast, in adipose tissue, only the high concentration of GE was able to induce changes in *fas*, *pparβ*, *gapdh*, and *lxr*. Moreover, in adipose tissue, the pattern of lipid metabolism-related genes expression induced by the high dose of GE presented a relevant degree of similarity with E2. In the liver, contrarily to white muscle and adipose tissue, the data pointed out the downregulation (blue) of *fas* in response to GE 5 μg/g while GE 50 μg/g increased *hsl*, as in the muscle. Moreover, the mRNA levels of the apoptosis markers analyzed were decreased or not altered in white muscle and adipose tissue, respectively. At the same time, in the liver, a differential response between GE doses could again be observed. Concerning autophagy-related genes, GE induced the strongest effects in white muscle and adipose tissue mostly at the high dose, while a weaker response was observed in the liver.

## 3. Discussion

Some studies have evaluated the effects of the dietary inclusion of soybean oil in replacement of FO in several fish species, such as sea bass, rainbow trout, or Atlantic salmon, in an attempt to decrease socioeconomic and environmental problems related to the increased pressure on marine ingredients [29,30,31,32,33]. However, there is still a lack of information about how its content in phytoestrogens (especially GE) could affect growth, metabolism, and fish health. In the current study, we evaluated the effects of GE and E2 on rainbow trout lipid metabolism and, apoptosis and autophagy regulation, using an in vitro culture of preadipocytes and an in vivo intraperitoneal injection as experimental models. 

Cell death has been mainly associated with three different processes: Apoptosis, cell death with autophagy, or necrosis [34], although autophagy is a complex phenomenon that can be both a cell death mechanism but also a protective response to stressful conditions [35]. In the present study, the lowest values of cell viability were found in GE 100 μM-treated adipocytes, together with the lowest NAF value, which is a useful early indicator of cell morphological changes occurring during the apoptotic process [28,36]. These effects did not seem to be induced through estrogen-like mechanisms, as values for both parameters were significantly different from those of the E2 group. On the other hand, preadipocytes exposed to the 100 μM dose of GE presented significantly higher protein levels of LC3-II compared to the other three groups. LC3b is a well-known specific marker for autophagy [37], the conserved process by which cytosolic components are trafficked to lysosomes for degradation [38]. During autophagy, LC3b is converted from a nonlipidated cytosolic form (LC3-I) to a lipidated and phosphatidylethanol-amine-conjugated one (LC3-II) [39]. Moreover, despite the low dose of GE did not show significant differences in the abovementioned parameters under the conditions tested, a longer GE 10 μM treatment, upregulated at a transcriptional level *lc3b*, and the lysosomal enzyme *ctsd*, while none of the apoptosis-related genes analyzed showed any differences in vitro. Results indicate that exposure to an acute high concentration of GE, or even a long treatment with a low dose, might induce cell death potentially due to increased autophagy in rainbow trout preadipocytes. In primary cultured human adipocytes, a very low dose of GE has been reported to increase cell viability, whereas high concentrations of this phytoestrogen decreased it [18]. Similarly, other studies performed in cell lines such as that of Gossner et al. [40] found a dose-dependent cytotoxic effect of GE on human ovarian carcinoma cells A2780, CaOV3, and ES2 through both apoptosis and autophagic mechanisms. The authors proposed that these results suggest a starvation-like signaling response, as cells could be rescued from apoptosis with methyl pyruvate [40]. A similar response in terms of apoptosis and cell death as a consequence of autophagy was also reported in MCF-7 breast cancer cells when exposed to GE 100 μM [41]. Likewise, high concentrations of GE also promoted developmental toxicity and increased mortality in zebrafish (*Danio rerio*) embryos [42,43,44]. Nevertheless, as far as we know, studies in fish cells have not been reported to date. Overall, the present in vitro results suggest that the cell death effect of GE in rainbow trout adipocytes could potentially be the consequence of an excessive stimulation of autophagy and not so clearly through an apoptotic process, as reported in mammalian models of obesity and diabetes [45], which is consistent both with the in vitro and in vivo gene expression data of autophagic and apoptotic markers. Nevertheless, the current data represent the first approach to understand the effects of GE on cell viability in this species, paving the way to the study of overall autophagic flux mechanisms in the future [39]. Moreover, we have to consider from a practical point of view that the induction of this response appears to be dose- and time-dependent; thus, an adequate low content of phytoestrogens (i.e., GE) in fish diets could be used to avoid this unfavorable effect.

Despite the decrease in cell viability, lipid accumulation, expressed in relation to protein, was increased by GE 100 μM and E2 treatments in rainbow trout preadipocytes, suggesting an adipogenic effect of GE at a high dose in this in vitro model, contrary to our initial hypothesis, which was based on mammalian literature on preadipocyte models [17,18]. Induction of cell differentiation in GE 100 μM and E2-treated cells also became evident by phenotypical changes, as cells acquired a round shape in addition to being filled with lipid droplets, characteristics of mature and hypertrophic adipocytes [46]. Adipogenesis is a multi-step process that includes the sequential activation of several transcription factors, such as CCAAT/enhancer binding protein alpha (C/EBPα) and PPARγ [14,47]. Furthermore, adipogenesis seems to be positively regulated by some autophagic-related genes, and indeed, the stability of PPARγ is increased by the process of autophagy in mammals [48], which is in agreement with the elevated levels of LC3-II observed in the cells exposed to GE 100 μM in the present work. The important role of autophagy during the whole process of adipogenesis in rainbow trout was also previously reported by our group [49]. There, the transcriptomic analysis revealed the coordinated expression of autophagy-related genes through the phases of proliferation and adipocyte maturation, showing an upregulation in each case of *atg5* and the lysosomal-associated membrane protein type 2A (*lamp2a*), respectively. Nevertheless, to our knowledge, information is not available about the possible pro-adipogenic and lipogenic in vitro effects of GE in fish species, nor about the involvement of autophagy in these processes.

In primary human adipocytes, Park et al. [18] found that GE inhibited lipid accumulation in a dose-dependent manner and, as a result of this, cells exposed to GE at a dose of 25 and 50 μM presented a fibroblast-like morphology. Similarly, GE decreased lipid accumulation in 3T3-L1 adipocytes, even at low concentrations of 25 μM [50]. The same anti-adipogenic effect has been reported through the decreased expression of some of the adipocyte differentiation-related genes (e.g., *cebpα*, *pparγ*, *fas*, *gapdh*) in the 3T3-L1 preadipocyte cell line [51] and human adipocytes [18]. Overall, these data suggested that GE may trigger a distinct regulation of cell differentiation between fish and mammals, being pro-adipogenic at least in rainbow trout. Nevertheless, the mechanisms underlying this differential regulation still have to be elucidated, but specific autophagic processes associated to cell differentiation might be involved. Notwithstanding, although most of the studies with mammalian preadipocytes have demonstrated an opposite (i.e., anti-adipogenic) response to GE, some have shown biphasic effects depending on the dose, mostly using mice or human bone marrow cultures [52], while in few cases, GE has been described as pro-adipogenic in a dose-dependent manner [53], in agreement with the current results.

With regard to E2, Haux and Norberg [54] reported elevated levels of TAG and phospholipids in the liver of rainbow trout after a weekly intraperitoneal injection of E2 in a 2 week trial. However, the effect on adipose tissue was not reported. In accordance with this, elevated hepatic lipogenesis and circulating cholesterol levels were also found in goldfish (*Carassius auratus*) after E2 exposure. Again, these are effects that differ from those observed in mammals, where low E2 levels seemed to promote lipid deposition in the liver [55]. This observation in fish agrees with the results of the present study, where the lipid content of the cells also increased with E2 treatment, indicating a pro-adipogenic effect. Furthermore, these data suggested that the specific GE effects on lipid accumulation described in rainbow trout adipocytes could be mediated through E2 receptors. However, studies with specific inhibitors would be needed to confirm this possibility.

On the other hand, in the present study, NEFA and glycerol levels released into the culture media did not show differences between treated and CT groups. These data are consistent with the absence of a lipolytic effect of GE, which, together with the increase in lipid accumulation as TAG caused by this phytoestrogen and E2, already discussed, indicates that both treatments are pro-adipogenic in rainbow trout adipocytes. 

Regarding the in vivo experiment, the administration of GE and E2 via intraperitoneal injection was selected as it offers the advantage of higher and faster exposure to the compounds than in administration via ingestion, as injected compounds will not suffer variations in the phase of absorption by the gastrointestinal tract, making it easier to elucidate their effects in the different tissues [56]. Adipose tissue, liver, and white muscle are described as the preferential lipid storage sites in teleost fish [46,57], and in the present work, the expression of lipid metabolism-related genes after GE injection revealed a differential regulation among them. Interestingly, GE at the dose of 50 μg/g and E2 elevated the expression of *vtg* in liver, a reliable indicator of estrogenic activity [58]. A positive correlation between E2 and the hepatic *vtg* expression is a well-established response in many fish species [58,59,60,61]. The upregulating effect of GE on *vtg* expression observed in this study was also reported in Atlantic salmon hepatocyte cells [24], and in the liver of rainbow trout [25]. Furthermore, plasma VTG levels also increased in rainbow trout after feeding a diet with a high content of phytoestrogens, including GE and daidzein [62]. With these results, we could suggest that GE can produce an estrogenic effect by acting as an E2-agonist in target tissues.

In adipose tissue, the mRNA levels of *fas* and *gapdh* were significantly upregulated in fish after 50 μg/g GE treatment compared to CT fish, clearly suggesting enhanced lipogenesis de novo, in line with the in vitro results observed regarding lipid accumulation. On the other hand, the increase in *pparβ* and *lxr* expression level may be an indication that the catabolism of fatty acids could also be activated at a certain degree [63,64]. By contrast, in the liver, GE decreased the gene expression of *fas* in parallel to upregulating *hsl*, indicating a lipolytic response. In white muscle, the balanced increase in lipogenesis and lipolysis markers indicated enhanced lipid turnover in this tissue, and the coordinated upregulation of both *pparα* and *pparβ* isoforms suggested an increased capacity for fatty acid oxidation. Contrarily to our study, and as mentioned in the in vitro discussion, GE generally induced the opposite response in mammalian adipose tissue. In a previous in vivo study by Naaz et al. [65], the administration of GE, via subcutaneous injection, decreased adipose tissue weight and the size of adipocytes in mice. Overall, in our study, the increased expression of lipogenesis markers in response to the high dose of GE may suggest a pro-obesogenic effect, which agrees with the increased lipid accumulation observed in cultured adipocytes, again rejecting our initial hypothesis. By contrast, our assumption applies for liver, where a lipolytic response was found, indicating a GE anti-obesogenic effect in this tissue; in this case, in agreement with the antisteatotic effect of GE described in humans, since hepatic TAG contents, LDL cholesterol, and liver weight have all been demonstrated to decline dramatically after a GE treatment [66]. In this aspect, apart from gene expression, the effects on the fish liver are not so well known. A previous study in liver of rainbow trout described a downregulation in *pparβ*, *pparγ*, and *rxr* transcription factors and increased expression of fatty acid synthesis-related genes such as *fas* in response to GE [25]. Therefore, in this species, FO replacement by soybean oil might lead to a dysregulation in lipid metabolism, causing a possible excess of lipid deposition, lowering the quality of the product. Nevertheless, in most cases, this response appeared to be induced only with a high dose of GE, suggesting again that it could be attenuated by adequately controlling the amount of this phytoestrogen incorporated in the diet.

In mammals, among its diverse properties (e.g., antioxidant, anti-inflammatory), GE has also been shown to induce apoptosis in different models like cancer cell lines [14,40,41,67,68], in rat brain [69], and in the adipose tissue of ovariectomized female mice [70], leading to consider this phytoestrogen as a potential therapeutic compound for several metabolic disorders, including obesity [14]. Furthermore, few studies have demonstrated that reduction in adipose tissue mass occurs not only as a result of stimulating lipolysis, but also by inducing cell apoptosis [71,72]. In mammals, and in contrast to GE, many studies have demonstrated the ability of E2 to inhibit or protect against the activation of apoptosis in many cell types [73,74]. Moreover, although most literature in mammals has reported the induction of apoptosis by GE, there are also references concerning the stimulation of autophagy coming from in vitro studies [75,76,77]. However, information about the anti- or pro-apoptotic effects of E2 in fish is still scarce [23,27]. In the present work, transcript levels of the apoptosis markers analyzed were reduced or not altered by GE in adipose tissue and white muscle. On the other hand, the effect on liver was not entirely clear, as *casp3* was upregulated with the high dose of GE, but *casp8* mRNA levels were decreased with GE at 5 μg/g concentration. This agrees with the in vitro results of this study, where the high dose of GE did not evidence a clear reduction in NAF. Similarly, Cleveland [23] found that *casp3* mRNA levels were downregulated in white muscle of rainbow trout after the administration of GE 5 μg/g, and also *casp9* with the 50 μg/g dose. Besides, daidzein, the second more important isoflavone found in soy, diminished gene expression of both *casp3* and *casp9*. In Senegalese sole at early life stages, evidence of induction of apoptosis by GE was not observed at transcriptional or protein levels, leading to normal larval development and growth [27]. Thus, unlike mammals, GE seems not to promote apoptosis in fish species, although further cellular assays should be performed to link them with gene expression data and confirm this hypothesis. Moreover, the effects observed in the apoptosis markers differ in direction and magnitude between GE and E2 in all the tissues evaluated, suggesting that, in this case, GE effects are mediated through estrogen receptor-independent mechanisms. 

Concerning autophagy-related genes, the high dose of GE induced an upregulating response in most of the genes analyzed, especially in the white muscle (*lc3b*, *atg4b*, and *atg12l*, although differences were not significant for the first one) and adipose tissue (*lc3b*, *atg4b,* and *ctsd*). In addition, the low dose of GE also enhanced the gene expression of *ctsd* and *ctsl* in white muscle. Moreover, the mRNA levels of *lc3b* showed in the adipose tissue the same pattern as that observed at gene and protein levels in cultured adipocytes in vitro, significantly increasing its expression with the GE 50 μg/g dose, thus supporting the enhanced macro-autophagic condition induced by GE in rainbow trout, which might lead to the cell death effect observed in vitro through the reduction in adipocyte viability values. Nonetheless, the obtained results do not allow deciphering whether GE, in addition to activating macro-autophagy, stimulates the chaperone-mediated autophagy (CMA). Until recently, the CMA process was thought to be restricted to mammals and birds [78], and, for this reason, it was not initially considered for the present study. However, a very recent work by Lescat and co-workers has shown that CMA also exists in fish [79], which opens new questions for future studies. Altogether, these data are in accordance with a previous study by Cleveland [23], which reported increased gene expression of multiple autophagy-related genes (e.g., *atg4b*, *lc3b*, *gabarapl1*, *ctsd*) in day 7 primary myocytes from rainbow trout exposed to a high dose of GE (100 μM). Indeed, as already mentioned, autophagy plays an important role in adipogenesis both in mammals and fish [38,49]. This pathway has been shown to regulate fat accumulation and PPARγ stability during adipogenesis, and to be essential for the cytoplasmic reorganization that occurs during adipocyte differentiation [38,45,48,49]. Moreover, studies in humans described an enhanced autophagic activity in adipose tissue from obese or type 2 diabetic individuals, through the increase in the autophagic flux and number of autophagosomes [80,81,82,83]. In fact, this observation agrees with the parallel lipogenic effect caused by GE in adipose tissue, both in vitro and in vivo, and in white muscle. Aside from that, the expression of *ctsd* has been demonstrated to be regulated by estrogen response elements [84] and, in a previous study, in rainbow trout performed by Cleveland and Weber [85], *ctsd* increased its expression in muscle in response to E2. In our study, this agrees with the highest gene expression of *ctsd* caused by E2 in the liver, but not in the other two tissues or in response to GE. Overall, these data indicate that GE, unlike E2, is able to modulate autophagic activity in fish, suggesting that these specific effects might be mediated by estrogen receptor-independent mechanisms.

## 4. Materials and Methods 

### 4.1. Animals and Ethics Statement 

Adult (in vitro experiment) and juvenile (in vivo experiment) rainbow trout, of approximately 80 and 250 g body weight, respectively, were obtained from the fish farm Trout Factory (Peramola, Lleida, Spain) and were acclimated for 15 days and maintained at the animal facilities (Spanish Operational Code REGA ES080/90036535) of the Faculty of Biology at the University of Barcelona (Spain). Animals were kept either in 0.2 or 0.4 m^3^ tanks under a 12 h light/12 h dark photoperiod with a temperature-controlled freshwater recirculation system at 18 ± 1 °C, and fed ad libitum twice daily with a commercial diet (Optiline-sf, Skretting, Burgos, Spain). Prior to sampling, juveniles were fasted from 2 h before the injection to 24 h post-injection (26 h), and adults for 24 h to avoid contamination from the gastrointestinal tract in the adipose tissue samples. Fish were anesthetized with MS-222 (0.1 g/l) (E10521, Sigma-Aldrich, Tres-Cantos, Spain) according to its effective dose in this species and sacrificed by a blow to the head before tissues sampling [86]. All animal handling procedures complied with the Ethics and Animal Care Committee of the University of Barcelona, in accordance with the guidelines of the European Union Council directive (2010/63 EU), and the Spanish and Catalan Government assigned principles and legislation (permit numbers CEEA OB35/17 and CEEA 311/15 for the in vitro and in vivo experiments, respectively).

### 4.2. Primary Culture of Preadipocytes and Experimental Treatments 

Primary cultures of rainbow trout preadipocytes were performed as previously described in Bouraoui et al. [87]. Briefly, after mechanical and enzymatic digestion of the tissue, the cells obtained were washed and counted. Then, cells were seeded at a final density of 2–2.5 × 10^4^ cells/cm^2^ in 1% gelatin-pretreated six-well plates (9.6 cm^2^/well) for Western blot and gene expression or twelve-well plates (2.55 cm^2^/well), with or without glass coverslips accordingly, for the viability assay, nuclear morphology analysis, ORO staining, and culture media parameters determination. Cells were cultured in Leibovitz’s L-15 medium (11415, Life Technologies, Alcobendas, Spain) supplemented with 10% fetal bovine serum and 1% of antibiotic-antimycotic solution (A5955, Sigma-Aldrich, Tres-Cantos, Spain) (growth medium, GM). The medium was changed every 2 days. Once confluence was reached (day 7), cells were induced to differentiate by means of incubation with a differentiation medium (DM) based on GM supplemented with 10 µg/mL insulin (I6634, Sigma-Aldrich, Tres-Cantos, Spain), 0.5 mM 3-isobutyl-1-methylxanthine (IBMX) (I5879, Sigma-Aldrich, Tres-Cantos, Spain), 0.25 µM dexamethasone (D4902, Sigma-Aldrich, Tres-Cantos, Spain), and 5 µl/mL lipid mixture (4.5 mg/mL cholesterol, 10 mg/mL cod liver oil, 25 mg/mL polyoxyethylenesorbitan monooleate, and 2 mg/mL D-α-tocopherolacetate) (L5146, Sigma-Aldrich, Tres Cantos, Spain). All plastic materials were obtained from Nunc (LabClinics, Barcelona, Spain).

GE (G6649, Sigma-Aldrich, Tres-Cantos, Spain) and E2 (E2758, Sigma-Aldrich, Tres-Cantos, Spain), used as a positive CT of the estrogenic effect, were applied at day 5 for 24 h to determine cell viability, nuclear morphology, and perform Western blots, or at day 7 for 72 h to measure biochemical parameters in culture media, lipid accumulation, and gene expression. GE and E2 were dissolved in dimethyl sulfoxide (DMSO), but the final concentration of DMSO added to the cells was 0.1% in all cases, which do not compromise cell integrity [28]. The treatments were the following: 1) CT (DMSO as vehicle), 2) GE 10 μM, 3) GE 100 μM, and 4) E2 1 μM. Doses were selected according to previous in vitro studies [18,23,41]. Four independent cultures were performed to perform Western blot (*n* = 4); six to assess cell viability, nuclear morphology, gene expression and biochemical parameters in culture media (*n* = 6); and twelve to analyze lipid accumulation (*n* = 12).

### 4.3. Cell Viability Assay 

The methylthiazolyldiphenyl-tetrazolium bromide (MTT) assay was used to evaluate cell viability. Briefly, pre-confluent adipocyte samples of two duplicate wells of the 12-well plates were incubated the last 18 h of the total 24 h treatment with a final concentration of 0.5 mg/mL of MTT (M5655, Sigma-Aldrich, Tres-Cantos, Spain). Next, cells were washed with PBS and the blue formazan crystals were allowed to resuspend in DMSO for 3 h. The viability values were obtained from the absorbance measured at 570 nm in duplicate 96-wells, with correction at 650 nm, using a microplate reader (Tecan Infinite M200, Männedorf, Switzerland). The value from cells containing PBS instead of MTT was also subtracted. Data are presented as a fold change relative to the CT group (*n* = 6 independent cultures). 

### 4.4. Nuclear Morphology Analysis 

NAF was used as a cell shape descriptor to assess apoptosis, as previously described [88]. After treatments incubation, cells attached to coverslips were fixed for 15 min with 4% paraformaldehyde in PBS containing 60 mM sucrose. After washing twice with PBS, and twice with PBS containing 20 mM glycine (PBS-glycine), permeabilization of cell membranes was done during 10 min with PBS-glycine supplemented with 0.05% saponin, and cell samples were subsequently rinsed with PBS. At this point, coverslips were blocked for 20 min with PBS-glycine containing 1% of BSA and then incubated for 15 min with 1% BSA and 0.025% saponin in PBS-glycine plus the DNA-specific fluorescent dye Hoechst 33342 (1 μg/mL) to stain the cell nuclei. Confocal images were obtained using a Leica TCS SP2 confocal microscope (Leica Microsystems, Mannheim, Germany) and processed with ImageJ software (National Institutes of Health, Bethesda, MD, USA). Per condition, ten random fields per coverslip were recorded and, per each culture, duplicate coverslips from 12-well plates were analyzed. NAF values were obtained from the product of nuclear area and roundness. All images were analyzed by the same researcher in a blinded manner (*n* = 4–6 independent cultures).

### 4.5. Western Blot 

The protein levels of the lipidated form of LC3 (LC3-II), a biomarker of autophagy, were analyzed by Western blot, following a previously published protocol [89], with slight modifications. Briefly, protein was extracted from cell samples of two duplicate wells from the 6-well plates with RIPA buffer (supplemented with proteases and phosphatases inhibitors). Samples were cooled on ice for 20 min and the supernatants were collected after a centrifuge at 12,000 rpm for 10 min. Then, quantification was done by the Bradford method and 2.7 μg of protein from each sample was subjected to sodium dodecyl sulfate polyacrylamide gel electrophoresis (SDS-PAGE) on 20% polyacrylamide gels containing 6 M urea (100 V for 2 h) and transferred to PVDF-FL membranes in transfer buffer (100 V and 75 min). At this point, membranes were blocked at RT for 1 h with an Odyssey^®^ Blocking buffer in TBS (Cat. No. 927-50000, Servicios Hospitalarios, Barcelona, Spain), and incubated overnight at 4 °C with rabbit polyclonal anti-LC3b antibody (1:300, Cat. No. 2775) and rabbit polyclonal anti-β-Tubulin (1:1000, Cat. No 2146), all from Cell Signaling Technology (Beverly, MA, USA), in the same blocking buffer. After washing 3 times with PBS-Tween 0.05%, membranes were incubated with a goat anti-rabbit fluorescence-conjugated secondary antibody (1:5000, Cat. No. 925-32211, Servicios Hospitalarios, Barcelona, Spain) for 1 h. Next, membranes were rewashed again, 3 times with PBS-Tween and twice with PBS, and the band signals were detected at 600 and 700 nm with the Odyssey^®^ FC Imaging System (LI-COR Inc. Biotechnology, Lincoln, NE, USA). Finally, the bands were quantified by Odyssey^®^ software Image Studio ver. 5.2.5. Data of LC3-II protein expression are presented as a fold change relative to each corresponding β-Tubulin protein expression (*n* = 4 independent cultures). 

### 4.6. Oil Red O Staining

Intracellular neutral lipid accumulation was analyzed by ORO staining, performed as in previous studies [28,90]. After 72 h treatments, cells were fixed for 1 h with 10% formalin and stained with 0.3% ORO (O0625, Sigma-Aldrich, Tres-Cantos, Spain) diluted in 36% triethyl phosphate during 2 h. After washing the excessive dye with distilled water and resuspending it in isopropanol, the quantification of the lipid content was calculated as the quotient of the absorbance measured at 490 nm by the one at 630 nm in duplicate 96-wells, using a microplate reader (Tecan Infinite M200, Männedorf, Switzerland). The reading at 630 nm corresponds to cell protein content, which was acquired by using the Coomassie brilliant blue G-250 dye for 1 h after finishing the ORO procedure, and extraction of the stain with propylene glycol during 1 h at 60 °C. Data are presented as the ratio of absorbance value between ORO and Coomassie blue staining (*n* = 12 independent cultures). The ORO staining effectiveness was assessed with a Zeiss Axiovert 40C (Carl Zeiss Inc., Oberkochen, Germany) inverted research-grade microscope equipped with a Canon EOS 1000D digital camera (magnification 20×).

### 4.7. Intraperitoneal Injection

After acclimation, juvenile fish were anesthetized (0.1 g/l MS-222) and received intraperitoneal injections at 4.64 μL volume per g body mass. Treatments were as follows: CT (DMSO diluted 1:3 in sesame oil (S3547, Sigma-Aldrich, Tres-Cantos, Spain) as this was the vehicle for the estrogenic compounds injections), GE at two doses, 5 and 50 μg/g body weight, and E2 at 5 μg/g (*n* = 6–9 fish). Doses were selected according to previous studies in rainbow trout, with the low one being similar to what is consumed on average by a fish fed with a soy-based diet, but not taking into account intestinal absorption/metabolism events [23,25]. After 24 h, animals were anesthetized (0.1 g/l MS-222) and blood samples were taken from the caudal vessels. After sacrifice by cranial concussion, samples of visceral adipose tissue, liver, and white muscle were collected, immediately frozen in liquid nitrogen, and stored at −80 °C until further analyses.

### 4.8. Biochemical Analysis of Culture Media and Plasma Metabolites

Glycerol and NEFA released into the cell culture media were measured for each experimental condition and culture (*n* = 6 independent cultures). Moreover, plasma glucose, NEFA, and TAG were analyzed in all sampled fish after the in vivo treatments (*n* = 6–9). All the procedures were done using commercial enzymatic kits (Wako Chemicals GmbH, Neuss, Germany; Spinreact, Sant Esteve d’en Bas, Spain and Monlab, Barcelona, Spain) following the manufacturers’ recommendations. 

### 4.9. Gene Expression Analyses

#### 4.9.1. RNA Extraction and cDNA Synthesis

Total RNA was extracted from adipose tissue (~100 mg), liver (~50 mg), and white muscle (~100 mg), or from cell samples of two duplicate wells of the 6-well plates using 1 mL of TRI Reagent (Applied Biosystems, Alcobendas, Spain), following the manufacturers’ recommendations. The quantity of isolated RNA was determined using a NanoDrop 2000 spectrophotometer (Thermo Fisher Scientific, Alcobendas, Spain), and integrity of the samples was confirmed in a 1% agarose gel (*w*/*v*) stained with SYBR-Safe DNA Gel Stain (Life Technologies, Alcobendas, Spain). In the case of the in vitro samples, enough RNA from the GE 100 μM condition could not possibly be obtained, because of the decrease caused in cell viability by this treatment, as explained in Section 2.1.1. Afterward, 1000 ng of total RNA was treated with DNase I (Invitrogen, Alcobendas, Spain) to remove all genomic DNA, and reverse-transcribed with the Transcriptor First Strand cDNA Synthesis Kit (Roche, Sant Cugat del Vallès, Spain).

#### 4.9.2. Real-Time Quantitative PCR (qPCR)

The mRNA transcript levels of the target genes plus three reference genes were examined in a CFX384^™^ real-time system (Bio-Rad, El Prat de Llobregat, Spain). Prior to the analyses, a dilution curve with a pool of samples was run to validate primer efficiency, specificity of the reaction, and absence of primer-dimers, as well as to determine the most appropriate cDNA working dilution required for each gene. Reactions were performed in triplicate (methodological replicates) using 384-well plates with 2.5 μL of iTaq Universal SYBR Green Supermix (Bio-Rad, El Prat de Llobregat, Spain), 250 nM of final concentration of forward and reverse primers (Table 3), and 1 μL of diluted cDNA for each sample, in a final volume of 5 μL. As described before [64], the protocol started with an initial activation step of 3 min at 95 °C, 40 cycles of 10 s at 95 °C, and 30 s at 54–62 °C (primer-dependent, see Table 3) followed by an amplicon dissociation analysis from 55 to 95 °C with a 0.5 °C increase each 30 s. The expression level of each gene evaluated was calculated with the Pfaffl method [91], relative to the mean of the two most stable reference genes, determined for each tissue by the geNorm algorithm implemented in the Bio-Rad CFX manager v. 3.1 software.

### 4.10. Statistical Analysis 

Data were analyzed using IBM SPSS Statistics v. 22 (IBM, Armonk, NY, USA) and plotted as mean + SEM with GraphPad Prism v. 7 (GraphPad Software, La Jolla, CA, USA, www.graphpad.com). Data normality and homoscedasticity were tested using Shapiro–Wilk and Levene’s tests, respectively. Statistical significance among groups was assessed by one-way analysis of variance (one-way ANOVA) followed by Tukey’s post-hoc test. When homoscedasticity was not observed, Dunnett’s T3 test was applied. Statistical differences were considered significant when the *p*-value was <0.05. 

## 5. Conclusions

In summary, the present work reports that GE at a high dose reduces cell viability and shows a tendency toward initiation of cell death in rainbow trout primary cultured preadipocytes, apparently mainly involving an enhanced stimulation of autophagy but not an apoptotic process, consistent with the gene expression changes observed both in vitro and in vivo. Nevertheless, caution is needed when the assumption is made that changes in transcript abundance are reflected in protein levels and/or enzyme activities, as numerous post-translational modifications and signaling pathways regulate these two processes. In addition, GE, in contrast to findings in mammals, induces adipogenic effects in the adipose tissue of this fish species, demonstrated through both in vitro and in vivo approaches. The positive correlation between GE and VTG, together with the similar responses between GE and E2 in lipid metabolism-related genes expression in adipose tissue and liver, suggest that this phytocompound can produce an estrogenic effect and act as an E2-agonist in some target tissues. On the whole, this study contributes to improving the knowledge on the impact of the dietary content of GE that can be found in some VO used for FO replacement in aquafeeds, and confirms the importance of testing and fine-tuning its amount on diets, as its effects are mainly dose-dependent. Consequently, an elevated dietary GE content (e.g., soy ingredients) would lead to adipose tissue growth and lipid metabolism impairment, mostly causing an excess in fat deposition in hypertrophic adipocytes, as a result of decreased cell viability, that could affect the quality of the aquaculture product.

## Figures and Tables

**Table 1:** Biochemical plasma parameters of rainbow trout intraperitoneally injected with genistein (GE) or 17β-estradiol (E2).

	CT	GE 5 μg/g	GE 50 μg/g	E2 5 μg/g
**TAG (mM)**	8.44 ± 1.14 ^a^	9.12 ± 1.83 ^a^	3.45 ± 0.81 ^b^	11.89 ± 2.38 ^a^
**NEFA (mM)**	0.151 ± 0.013 ^a^	0.182 ± 0.012 ^a^	0.156 ± 0.006 ^a^	0.250 ± 0.038 ^b^
**Glucose (mM)**	6.08 ± 0.46	6.37 ± 0.50	4.39 ± 0.73	5.96 ± 0.85

Plasma levels of triacyclglycerols (TAG), non-esterified fatty acids (NEFA), and glucose in rainbow trout intraperitoneally injected with vehicle (DMSO diluted 1:3 in sesame oil) as CT, GE 5 μg/g body weight, GE 50 μg/g, or E2 5 μg/g. Data are shown as mean ± SEM (*n* = 4–9). Significant differences among treatments were determined by one-way ANOVA and are indicated by different letters (a, b) (*p* < 0.05).

**Table 2:** Gene expression of hepatic vitellogenin (*vtg*) in rainbow trout intraperitoneally injected with genistein (GE) or 17β-estradiol (E2).

	CT	GE 5 μg/g	GE 50 μg/g	E2 5 μg/g
***vtg***	0.196 ± 0.041 ^a^	0.168 ± 0.028 ^a^	0.653 ± 0.100 ^b^	1.362 ± 0.131 ^c^

Relative mRNA expression of *vtg* normalized to *ubq* and *β-actin* in rainbow trout intraperitoneally injected with vehicle (DMSO diluted 1:3 in sesame oil) as CT, GE 5 μg/g body weight, GE 50 μg/g, or E2 5 μg/g. Data are shown as mean ± SEM (*n* = 6–9). Significant differences among treatments were determined by one-way ANOVA and are indicated by different letters (a, b, c) (*p* < 0.05).

**Table 3:** Primers used in the real-time quantitative PCR analyses.

Gene	Primer Sequences (5′–3′)	Tm, °C	Accession Number
*ubq*	F: ACAACATCCAGAAAGAGTCCA R: AGGCGAGCGTAGCACTTG	58	NM_001124194.1
*ef1α*	F: TCCTCTTGGTCGTTTCGCTG R: ACCCGAGGGACATCCTGTG	58	NM_001124339.1
*β-actin*	F: ATCCTGACGGAGCGCGGTTACAGC R: TGCCCATCTCCTGCTCAAAGTCCA	61	AJ438158
*vtg*	F: GAGCTAAGGTCCGCACAATTG R: GGGAAACAGGGAAAGCTTCAA	58	X92804
*fas*	F: GAGACCTAGTGGAGGCTGTC R: TCTTGTTGATGGTGAGCTGT	54	tcaa0001c.m.06_5.1.om.4
*lpl*	F: TAATTGGCTGCAGAAAACAC R: CGTCAGCAAACTCAAAGGT	59	AJ224693
*hsl*	F: AGGGTCATGGTCATCGTCTC R: CTTGACGGAGGGACAGCTAC	58	TC172767
*pparα*	F: CTGGAGCTGGATGACAGTGA R: GGCAAGTTTTTGCAGCAGAT	54	NM_001197211.1
*pparβ*	F: CTGGAGCTGGATGACAGTA R: GTCAGCCATCTTGTTGAGCA	60	AY356399.1
*gapdh*	F: TCTGGAAAGCTGTGGAGGGATGGA R: AACCTTCTTGATGGCATCATAGC	61	NM_001123561
*lxr*	F: TGCAGCAGCCGTATGTGGA R: GCGGCGGGAGCTTCTTGTC	62	NM_001159338
*casp3*	F: TTTGGGAGTAGATTGCAGGG R: TGCACATCCACGATTTGATT	57	TC139042
*casp8*	F: CAGCATAGAGAAGCAAGGGG R: TGACTGAGGGGAGCTGAGTT	56	TC172513
*p53*	F: GTGGAATTTGATCCGAGTCTGT R: AGTGTCCAGGGTAGAATGGAG	56	-
*lc3b*	F: GAACAGTTTGACCTGCGTGAA R: TCTCTCAATGATGACCGGAATCT	57	CA350545
*atg4b*	F: TATGCGCTTCCGAAAGTTGTC R: CAGGATCGTTGGGTTTCTGC	58	CA345181.s.om.10
*atg12l*	F: GATGGAGGCCAATGAACAGC R: GCGTTTGAACTGAAAAGGGCTAA	60	CB490089.s.om.10
*ctsd*	F: GCCTGTCATCACATTCAACT R: CCACTCAGGCAGATGGTCTTA	55	NM_001124711
*ctsl*	F: TGAAGGAGAAGATGTGGATGG R: TTCCTGTCTTTGGCCATGTAG	56	NM_001124305

F: Forward; R: Reverse; Tm: Melting temperature; *ubq*: Ubiquitin; *ef1α*: Elongation factor 1 alpha; *β-actin*: Beta-actin; *vtg*: Vitellogenin; *fas*: Fatty acid synthase; *lpl*: Lipoprotein lipase; *hsl*: Hormone sensitive lipase; *pparα*: Peroxisome proliferator-activated receptor alfa; *pparβ*: Peroxisome proliferator-activated receptor beta; *gapdh*: Glyceraldehyde-3-phosphate dehydrogenase *lxr*: Liver x receptor; *casp3*: Caspase 3; *casp8*: Caspase 8; *p53*: Tumor protein p53; *lc3b*: Microtubule-associated protein-1 light chain 3b; *atg4b*: Autophagy-related 4b cysteine peptidase; *atg12:* Autophagy-related gene 12-like; *ctsd*: Cathepsin d; *ctsl*: Cathepsin l.

## Abbreviations

*atg*	Autophagy-related gene
*casp*	Caspase
CT	Control
CMA	Chaperone-mediated autophagy
*cts*	Cathepsin
DMSO	Dimethyl sulfoxide
*ef1α*	Elongation factor 1 alpha
E2	17β-Estradiol
*fas*	Fatty acid synthase
FO	Fish oil
*hsl*	Hormone-sensitive lipase
*gapdh*	Glyceraldehyde-3-phosphate dehydrogenase
GE	Genistein
LC3b	Microtubule-associated protein-1 light chain 3b
*lpl*	Lipoprotein lipase
*lxr*	Liver x receptor
MTT	Methylthiazolyldiphenyl-tetrazolium bromide
NAF	Nuclear area factor
NEFA	Non-esterified fatty acids
ORO	Oil Red O staining
*ppar*	Peroxisome proliferator-activated receptor
*p53*	Tumor protein p53
TAG	Triglycerides
*ubq*	Ubiquitin
VO	Vegetable oil
*vtg*	Vitellogenin
